# Web-Based Warfarin Management (Alfalfa App) Versus Traditional Warfarin Management: Multicenter Prospective Cohort Study

**DOI:** 10.2196/46319

**Published:** 2024-07-29

**Authors:** Wenfei Chen, Jiana Chen, Shaojun Jiang, Chunhua Wang, Jinhua Zhang

**Affiliations:** 1 Department of Pharmacy Fujian Maternity and Child Health Hospital College of Clinical Medicine for Obstetrics & Gynecology and Pediatrics Fujian Medical University Fuzhou China; 2 Department of Neurosurgery Fujian Medical University Union Hospital fuzhou China

**Keywords:** warfarin, telemedicine, smart phone application, anti-coagulation management, management, cohort study, application, chronic disease, support, effectiveness, online model, patient management

## Abstract

**Background:**

Poor anticoagulation management of warfarin may lead to patient admission, prolonged hospital stays, and even death due to anticoagulation-related adverse events. Traditional non–web-based outpatient clinics struggle to provide ideal anticoagulation management services for patients, and there is a need to explore a safer, more effective, and more convenient mode of warfarin management.

**Objective:**

This study aimed to compare differences in the quality of anticoagulation management and clinical adverse events between a web-based management model (via a smartphone app) and the conventional non–web-based outpatient management model.

**Methods:**

This study is a prospective cohort research that includes multiple national centers. Patients meeting the nadir criteria were split into a web-based management group using the Alfalfa app or a non–web-based management group with traditional outpatient management, and they were then monitored for a 6-month follow-up period to collect coagulation test results and clinical events. The effectiveness and safety of the 2 management models were assessed by the following indicators: time in therapeutic range (TTR), bleeding events, thromboembolic events, all-cause mortality events, cumulative event rates, and the distribution of the international normalized ratio (INR).

**Results:**

This national multicenter cohort study enrolled 522 patients between June 2019 and May 2021, with 519 (99%) patients reaching the follow-up end point, including 260 (50%) in the non–web-based management group and 259 (50%) in the web-based management group. There were no observable differences in baseline characteristics between the 2 patient groups. The web-based management group had a significantly higher TTR than the non–web-based management group (82.4% vs 71.6%, *P*<.001), and a higher proportion of patients received effective anticoagulation management (81.2% vs 63.5%, *P*<.001). The incidence of minor bleeding events in the non–web-based management group was significantly higher than that in the web-based management group (12.1% vs 6.6%, *P*=.048). Between the 2 groups, there was no statistically significant difference in the incidence of severe bleeding and thromboembolic and all-cause death events. In addition, compared with the non–web-based management group, the web-based management group had a lower proportion of INR in the extreme subtreatment range (17.6% vs 21.3%) and severe supertreatment range (0% vs 0.8%) and a higher proportion in the treatment range (50.4% vs 43.1%), with statistical significance.

**Conclusions:**

Compared with traditional non–web-based outpatient management, web-based management via the Alfalfa app may be more beneficial because it can enhance patient anticoagulation management quality, lower the frequency of small bleeding events, and improve INR distribution.

## Introduction

With a usage history spanning over 60 years, warfarin is currently the most widely used anticoagulant prescription drug for preventing thrombosis after heart valve surgery, orthopedic surgery, atrial fibrillation, and varicose veins of the lower extremities, as well as systemic embolism, pulmonary embolism, and venous thromboembolism [1]. Daily management of warfarin is highly correlated with clinical outcomes. High-quality anticoagulation management can effectively reduce the risk of bleeding and thromboembolic events during dosing, while poor anticoagulation management may lead to hospital admission, prolonged hospital stay, and even patient death due to anticoagulation-related adverse events. The anticoagulant intensity of warfarin is measured by the international normalized ratio (INR), with different target ranges of INR for different anticoagulation indications. Warfarin's intensity of anticoagulation can be influenced by numerous variables. Along with the generally constant elements like genotype and gender, other variables may evolve with time, resulting in modifications in the pharmacokinetic and pharmacodynamic properties of warfarin in addition to changes in INR. At the same time, patients who take warfarin need to have INR testing done regularly and receive personalized dose adjustments to maintain the proper anticoagulant intensity owing to the drug's limited therapeutic window, the numerous factors that can influence its anticoagulant effect, and the wide range of individual differences. [2].

Currently, the quality of anticoagulation in Chinese patients receiving warfarin therapy is not satisfactory [3]. On the one hand, due to the relative scarcity of medical and health resources, as well as their unequal geographical distribution in China [4], physicians and pharmacists with specialized anticoagulation training are usually concentrated in urban areas, and patients in most rural areas lack convenient access to high-quality anticoagulation management. On the other hand, the traditional non–web-based management model requires patients to have good compliance, to be able to take their medication on schedule, and to be proactive in visiting the hospital regularly for INR testing and dose adjustment. However, in clinical practice, patients often miss medication and prolong the interval of blood examination by themselves. All these factors increase the risk of warfarin-related anticoagulant complications [5].

Therefore, it is necessary to explore a safer, more effective, and convenient mode of warfarin management for patients. We have previously conducted a retrospective study in a single center [3], but there are often issues with retrospective studies, such as low data completeness and accuracy, which may introduce selective bias. Therefore, we conducted a nationwide multicenter prospective cohort study to collect patient demographic information, coagulation test results, and clinical events and further compare whether there are any differences in the quality of anticoagulation management and cardiovascular event rates between web-based management provided through a smartphone app and traditional non–web-based outpatient management.

## Methods

### Study Design and Participants

**Figure 1:**
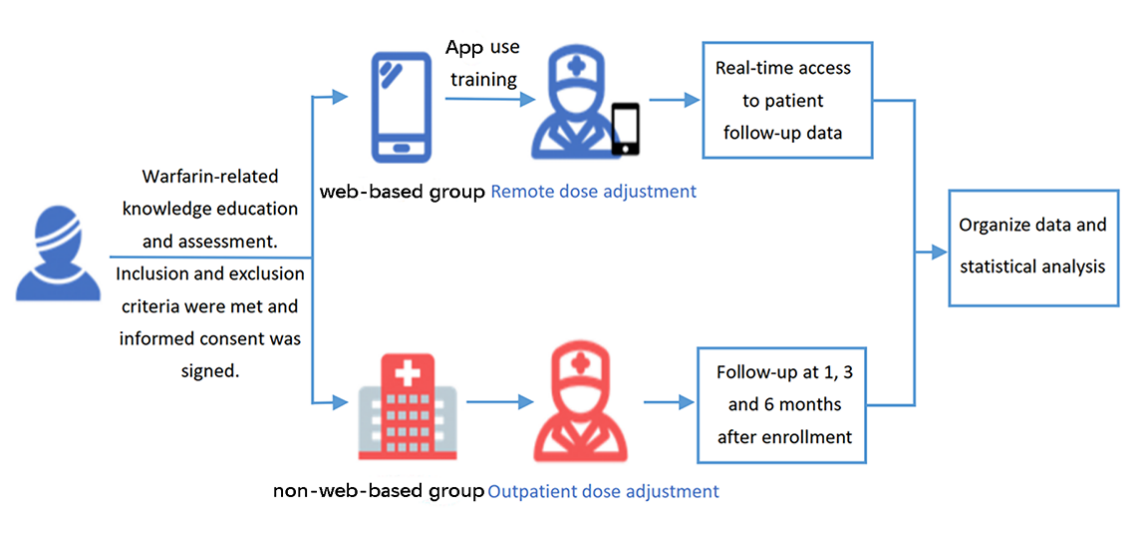
Flowchart of the study.

We conducted a national multicenter prospective cohort study to explore the differences in quality of anticoagulation management as well as clinical adverse events between the web-based management model via the Alfalfa app and the traditional non–web-based outpatient management model. The study flow is shown in Figure 1. This study was reported in strict adherence to the RECORD-PE (Reporting of Studies Conducted Using Observational Routinely Collected Health Data Statement for Pharmacoepidemiology) checklist (Multimedia Appendix 1) [6]. Participants were enrolled at 5 central hospitals in China from June 2019 to May 2021, after approval by the ethical committees of each center. These 5 hospitals are in the southwest, southeast, and central parts of China, which are different in terms of geographic location, climate, and diet, and to a certain extent represent most parts of the country and are representative. The study was also registered with the Chinese Clinical Trial Registry (ChiCTR1900021920). At the time of registration, this study was designed as a randomized controlled trial, but during actual grouping, it was difficult to carry out randomized grouping because the patients were in the same ward and their desire to group on their own was so strong that it could only be designed as a prospective cohort study in the end. The associated study design has been previously published [7].

### Ethical Considerations

This study was approved by the Ethics Committee of Union Hospital affiliated with Fujian Medical University (2019YF020-01). The local ethical committee of each hospital also provided ethics approval. All patients had to sign an informed consent form to participate.

### Inclusion and Exclusion Criteria

The inclusion criteria were: (1) age ≥18 years; (2) patients with the following indications and requiring warfarin therapy for at least 3 months: receiving heart valve replacement or repair, atrial fibrillation confirmed by electrocardiogram or ambulatory electrocardiogram, receiving radio-frequency/cryoballoon ablation, and venous thromboembolism confirmed by venography or Doppler ultrasound; (3) willingness to learn to use and accept web-based or non–web-based warfarin administration; (4) willingness to be followed up; and (5) willing to sign patient consent and signed informed consent form.

To address potential sources of bias, we excluded patients who (1) were pregnant or planning to become pregnant; (2) taking or planning to take other oral anticoagulants; (3) had experienced therapeutic or subtherapeutic bleeding or thrombotic complications in the 6 months prior to enrollment; (4) expected to have an imminent procedure requiring warfarin interruption; (5) had severe renal insufficiency (endogenous creatinine clearance ≤20 mL/min); (6) had severe hepatic insufficiency (Child-Pugh score ≥ 10); (7) had severe heart failure (New York Heart Association heart function class IV and above); and (8) had severe infection and respiratory failure.

### Patient Management

Patients in the web-based management group were managed remotely via a smartphone app, where the investigator provided dose adjustment protocols and had immediate access to patient follow-up data. The web-based management tool used in this study was the Alfalfa app (Fuzhou Alfalfa Health Management Co). The non–web-based management group was defined as patients who used the usual outpatient route for dose adjustment after being discharged from the hospital. The investigators plan to follow up with patients in the non–web-based management group at 1, 3, and 6 months after enrollment to collect coagulation test results and data related to clinical events.

### Grouping and Pregrouping Education

For all patients who met the inclusion and exclusion criteria, the investigators provided between 3 and 4 warfarin dosing information sessions to the patients and their relatives prior to discharge and distributed electronic dosing education materials. The materials went over the effects of warfarin, medication purpose, medication precautions, indicators to be monitored during administration and their importance, the INR target range, the INR monitoring interval, factors affecting the efficacy of warfarin, and the potential side effects of warfarin.

Patients in the non–web-based management group were instructed by the investigator to attend the nearby hospital or clinic frequently for coagulation testing and to consult with their local physician for dose adjustment options. Patients and their relatives were also instructed to keep track of the results and dates of all previous INR tests, as well as any possible bleeding or thrombotic events that occurred during this period. Finally, patients were notified that they would be followed up at 1, 3, and 6 months with the expectation of patient cooperation.

Patients in the web-based management group were provided with extra training on operating the Alfalfa app, which focused on registering the software, main functions, procedures to upload coagulation results, and instructions on taking medication and checking blood according to the medical staff's response. The investigators ensured that patients or their relatives were able to operate the Alfalfa app independently and accurately.

### Anticoagulation Management Program

#### INR Target Range

The INR target ranges for each anticoagulation indication in this study are shown in Table 1. If the patient had a combination of multiple anticoagulation indications, a higher INR target range corresponding to the indications was used in the development of the dose adjustment protocol. It is worth noting that the target range of INR after heart valve surgery was based on clinical experience at the main central hospital and is not entirely consistent with the target range recommended by the Chinese Guidelines for the Prevention and Treatment of Thrombotic Diseases. This INR target range has been applied to over 40,000 patients for more than 30 years and can ensure the security and efficacy of anticoagulation therapy.

**Table 1:** INR^a^ target ranges for different indications.

Anticoagulation indication	INR target range
Aortic valve replacement or repair	1.5-2
Mitral valve replacement or repair	1.7-2.5
Tricuspid valve replacement or repair	2-2.5
Atrial fibrillation	2-3
Venous thromboembolism	2-3

^a^INR: international normalized ratio.

#### INR Monitoring Frequency

Each patient’s initial INR was checked weekly after discharge. When 2 consecutive INR values were within the patient's target range, their INR testing interval could be extended by 1 week (ie, once every 2 weeks). With biweekly INR values, if 2 consecutive INR values were within the patient's target range, the blood test interval could be extended by 1 week (ie, every 3 weeks), and so on. The maximum interval between blood tests could not exceed 1 month (ie, the INR value should be tested at least once a month). If the warfarin dose needed adjustment, the process returned to weekly blood checks and was repeated until the dose stabilized again. If a patient reported extreme INR values, the anticoagulation management team planned to empirically adjust the INR monitoring interval based on previous INR records.

#### Dose Adjustment Scheme

The dose adjustment protocol for warfarin is empirically developed by clinical pharmacists or physicians based on information such as INR results, comorbidities and medications, and clinical events with reference to the Chinese Expert Consensus on Warfarin Anticoagulation Therapy. The general principles of dose adjustment are as follows：(1) if the INR value is within ± 0.2 of the upper and lower limits of the target range, the warfarin dose remains unchanged; (2) if INR <lower limit of the target range, 0.2, warfarin increases by 0.25 tablets; (3) if the upper limit of target range + 0.2 <INR ≤3, warfarin decreases by 0.25 tablets; (4) If INR >3, stop the drug for 1 day and recheck INR on the next day; (5) if INR ≤3 on the next day, reduce 0.25 tablets and repeat the aforementioned process; and (6) if INR >3 on the next day, continue to stop the drug until INR ≤3.

#### Management Style

Patients in the web-based management group were managed remotely with the Alfalfa app based on the WeChat public platform for anticoagulation (see Multimedia Appendix 2 [8-12] for details on the Alfalfa app). The anticoagulation management team at each central hospital consists of clinical pharmacists and physicians specializing in anticoagulation. They were responsible for providing dose adjustment and medication counseling to patients in the web-based management group. Web-based management was achieved through the following steps. First, the patient patients had to inform the physician or pharmacists of the results of the coagulation report as well as recent physical and dietary conditions, concomitant medications, adverse reactions, and other information. Second, the anticoagulation management team members responded to the patient with the recommended dose and time of the next blood test based on the information reported by the patient. Finally, patients took their medication as prescribed and performed their next coagulation test on time. If a patient reported an extreme INR value or clinical event, the medical staff followed up with the patient by phone to ask about their medical condition and advise on the proper management. Patients in the non–web-based management group underwent dose adjustments at their local hospital, with specific anticoagulation management protocols developed by their local physician or pharmacist.

### Outcome Indicators

The time in therapeutic range (TTR), this study’s primary outcome metric, was calculated using the linear interpolation method proposed by Professor Rosendaal. The secondary outcome indicators were the occurrence of clinical events and the distribution of INR. The clinical event focused on evaluating the differences in safety and effectiveness between web-based and traditional non–web-based outpatient management. Safety outcomes referred to bleeding events and were classified as minor bleeding events and serious bleeding events. The former included epistaxis, gingival or oral bleeding, skin ecchymosis, fundus hemorrhage, menorrhagia or prolongation, and hematuria. Using the classification criteria of the International Society on Thrombosis and Hemostasis (ISTH) [13], the latter was defined as any hemorrhage requiring hospitalization or blood transfusion, including gastrointestinal bleeding and intracranial hemorrhage. Effective outcomes were thromboembolic events, including transient ischemic attack, venous thromboembolism, valve thrombosis, and ischemic stroke. Other clinical events included hospitalizations and emergencies associated with warfarin. Likewise, the distribution of INR was used as part of the secondary outcome indicators in this study. The patients' INR outcomes were classified as extreme subtherapeutic range, subtherapeutic range, within therapeutic range, supertherapeutic range, and extreme supertherapeutic range according to their INR target range. Table 2 shows the specific definitions of these 5 ranges.

**Table 2:** Classification criteria for INR^a^.

INR classification	Definition
Extreme subtherapeutic range	INR < lower limit of target range (0.2)
Subtherapeutic range	Lower limit of target range (0.2) ≤ INR < lower limit of target range
Therapeutic range	Lower limit of target range ≤ INR ≤ upper limit of target range
Supratherapeutic range	Upper limit of target range＋0.2< INR ≤4.5
Extreme supratherapeutic range	INR >4.5

^a^INR: international normalized ratio.

### Sample Size Calculation

This study used the primary outcome indicator TTR to calculate the sample size. Based on the results of previous studies, the TTR was approximately 73.1% and 66.% for the web-based and non–web-based groups, respectively [14]. When the one-class error (α) was .05 and the degree of certainty (1-β) was 80%, the web-based group was included in a 1:1 ratio to the non–web-based group, and a minimum of 206 patients per group was required as calculated by the statistical software Power Analysis & Sample Size (version 15; NCSS). Assuming a 20% lost-to-review rate, 258 patients were needed in each group, for a total of 516 patients.

### Data Collection and Management

All patients’ demographic information was collected from the hospital information system. INR records and patients’ clinical events in the non–web-based management group were obtained by telephone follow-up, which was conducted at 1, 3, and 6 months after patient enrollment. Patients who did not meet the follow-up end point were excluded. INR records and clinical events of patients in the web-based management group were derived from the Alfalfa app background management system.

### Statistical Analysis

The Kolmogorov-Smirnov test was used to verify the normality of the distribution of all continuous variables. Normally distributed continuous variables were expressed as mean (SD), and the Student *t* test was used to compare the differences between the 2 groups of variables. Continuous variables that did not conform to the normal distribution are represented as medians (IQR), and their differences were compared by using the Mann-Whiney U test. Categorical variables were represented as examples (percentages), and chi-square tests or Fisher exact probability methods were used for the statistical analysis of categorical variables. In addition, the difference in the cumulative event rate between the 2 groups was represented by a Kaplan-Meier curve and compared by a log-rank test. In this study, the difference was statistically significant when *P*<.05. The aforementioned statistical analysis was performed using SPSS software (version 20.0; IBM Corp) and R software (version 4.1.1; R Foundation for Statistical Computing).

## Results

### Baseline Situation

A total of 522 patients were enrolled in this study between June 2019 and May 2021, with the last follow-up completed on November 12, 2021. Among all patients, 3 (0.57%) in the non–web-based group did not meet the follow-up end point because 1 (0.19%) patient became pregnant during follow-up and 2 (0.38%) patients refused follow-up. The final data analysis included information on a total of 519 patients, including 260 web-based patients (50%) and 259 non–web-based patients (50%). The patients’ baseline information is shown in Table 3.

Among the patients in the non–web-based management group, 140 (53.8%) were male. The average age was 49.1 (SD 11.7) years, and the average BMI was 22.1 (SD 4.1) kg/m^2^. The number of patients who smoked and drank was 79 (30.4%) and 55 (21.2%), respectively. In the web-based management group, there were 155 (59.9%) male patients, the average age was 48 (SD 14) years, and the average BMI was 22.6 (SD 3.9) kg/m^2^. The number of patients who smoked and drank was 69 (26.6%) and 60 (23.2%), respectively. There was no statistically significant difference in demographic information between the 2 groups (*P*>.05). The most common comorbidity, which accounted for more than 15% (n=40) of patients in both groups, was hypertension. The proportion of patients with antiplatelet therapy was 3.9% (n=10) in the web-based management group, slightly higher than the 1.6% (n=3) in the non–web-based management group, but the difference was not statistically significant. Except for antiplatelet therapy, the 2 groups were similar in terms of other comorbidities and medications, and neither group showed a statistically significant difference. In addition, there was no statistically significant difference in the proportion of patients with each anticoagulant indication between the 2 groups.

**Table 3:** Baseline information of the study population.

Variable name			Non–web-based management group (n= 260)		Web-based management group (n=259)	*P* value
**Demographic information**						
	Male sex, n (%)	140 (53.8)		155 (59.9)		.18
	Age (years), mean (SD)	49 (11.7)		48 (14)		.40
	BMI (kg/m^2^), mean (SD)	22.1 (4.1)		22.6 (3.9)		.12
	Smoking history, n (%)	79 (30.4)		69 (26.6)		.38
	Drinking history, n (%)	55 (21.2)		60 (23.2)		.60
**Concomitant diseases and medications, n (%)**						
	Hypertension	44 (16.9)		40 (15.4)		.72
	Diabetes	12 (4.6)		14 (5.4)		.69
	Gout or hyperuricemia	7 (2.7)		11 (4.3)		.35
	Combined antiplatelet therapy	3 (1.6)		10 (3.9)		.05
	Previous history of thrombosis	7 (2.7)		9 (3.5)		.62
	Previous history of bleeding	3 (1.2)		2 (0.8)		.99
	Combined tumor	0 (0)		2 (0.8)		.25
	Combined atrial fibrillation	1 (0.4)		0 (0)		.99
**Anticoagulation indications, n (%)**						
	Heart valve replacement or repair	251 (96.5)		252 (97.3)		.80
	Atrial fibrillation	5 (1.9)		2 (0.8)		.45
	VTE^a^	4 (1.6)		5 (1.9)		.75

^a^VTE: venous thromboembolism.

### Anticoagulation Management Quality

**Figure 2:**
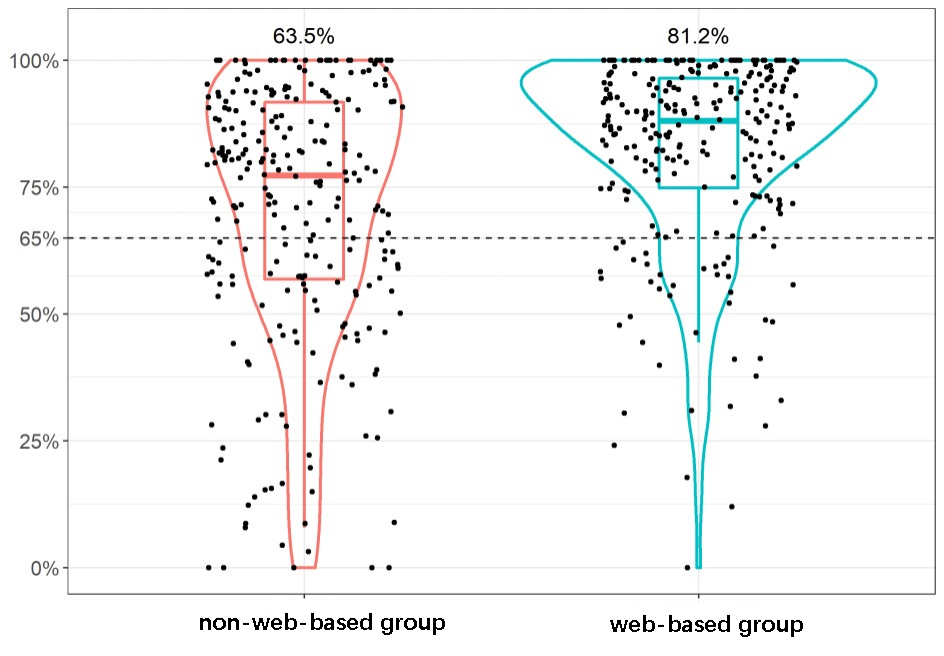
Time distribution in the treatment range.

In this study, a total of 6084 INR records were collected, including 3125 times (51.4%) in the non–web-based management group and 2959 times (48.6%) in the web-based management group. The TTR of the non–web-based management group was significantly lower than in the web-based management group, at 71.6% (SD 25.9%) versus 82.4% (SD 18.6%), respectively (*P*<.001). The distribution of TTR is shown in Figure 2. The proportion of TTR greater than 65% in the non–web-based management group was significantly lower than in the web-based management group, and there was a statistical difference between the 2 groups (63.5% vs 81.2%, *P*<.001).

The distribution of INR values is shown in Table 4. The proportion of INR values in the non–web-based management group in the extreme subtreatment range and extreme overtreatment range was significantly higher than that in the web-based management group, and the proportion in the treatment range was significantly lower than that in the web-based management group (*P*<.001). However, the proportion of INR values in the subtherapeutic range and the supertherapeutic range in the non–web-based management group was also higher than that in the web-based management group, but the difference was not statistically significant (*P*=.18 and *P*=.14, respectively).

**Table 4:** The distribution of INR^a^ values.

INR value classification	Non–web-based management group (n=3125), n (%)	Web-based management group (n=2959), n (%)	*P* value
Extreme subtherapeutic range	666 (21.3)	521 (17.6)	<.001
Subtherapeutic range	515 (16.5)	450 (15.2)	.18
Therapeutic range	1348 (43.1)	1490 (50.4)	<.001
Supratherapeutic range	572 (18.3)	498 (16.8)	.14
Extreme supratherapeutic range	24 (0.8)	0 (0)	<.001

^a^INR: international normalized ratio.

### Clinical Event Outcomes

**Figure 3:**
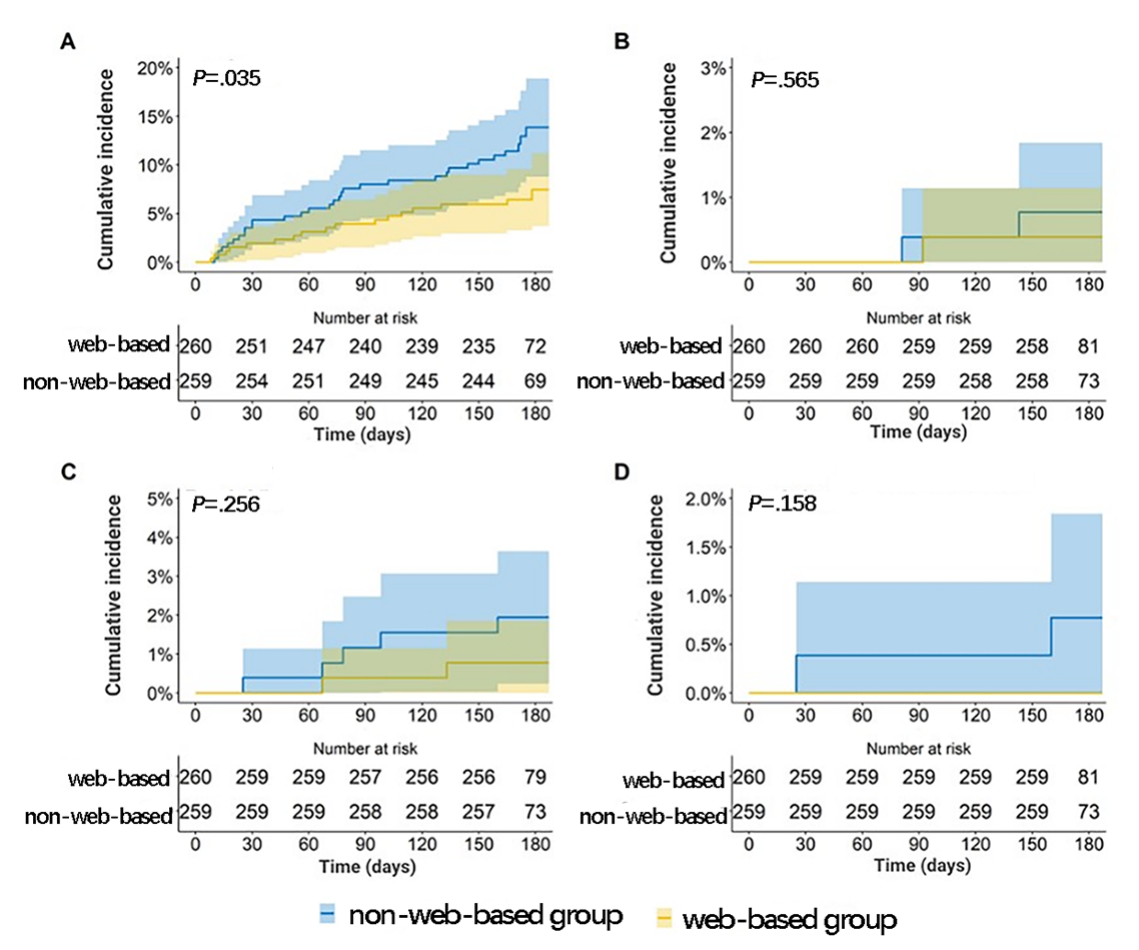
Kaplan-Meier curve. A. Minor bleeding; B. Severe bleeding; C. Thromboembolic; D. All-cause deaths.

In terms of clinical events, the non–web-based management group had a significantly higher incidence of minor bleeding events than the web-based management group (12.1% vs 6.6%, respectively, *P*=.048). Furthermore, there was no significant difference in the incidence of major bleeding events and thromboembolic events between the 2 groups. A total of 2 (0.78%) fatalities were observed in patients in the non–web-based management group, with acute prosthetic valve thrombosis and acute ischemic stroke. There were no recorded fatalities in the web-based management group. The specific occurrence of clinical events is shown in Multimedia Appendix 3. Epistaxis and gingival bleeding were the 2 most common types of bleeding, with a total of 16 (6.18%) and 19 (7.33%) cases, respectively. Figure 3 shows the cumulative clinical event rate between the web-based management group and the non–web-based management group during the follow-up period. Compared with the web-based management group, the non–web-based management group had a higher cumulative incidence of minor bleeding time (*P*=.035), but there were no statistical differences in severe bleeding time, thrombotic events, and all-cause mortality between the 2 groups (*P*>.05). Table 5 shows the clinical event outcomes.

**Table 5:** Clinical event outcomes.

Clinical events	Non–web-based management group (n= 260), n (%)	Web-based management group (n= 259), n (%)	*P* value
Minor bleeding events	31 (12.1)	17 (6.6)	.048
Severe bleeding events	2 (0.78)	1 (0.39)	.99
Thromboembolic events	5 (1.95)	2 (0.77)	.45
All-cause death events	2 (0.78)	0 (0)	.49

## Discussion

### Principal Findings

This national multicenter prospective cohort study is the first in China to compare the difference between the quality of anticoagulation management and clinical event outcomes between web-based anticoagulation management carried out via the smartphone Alfalfa app and traditional non–web-based outpatient management. The study was conducted in 5 centers in China, located in the southwest, southeast, and central regions, which are disparate in terms of geographical location, climate, and diet. To some extent, these regions represent most of the country. In general, a TTR greater than 65% is considered the standard for effective anticoagulation management, in which case the benefits of anticoagulation outweigh the bleeding risk associated with anticoagulation [15]. The average TTRs of patients in the non–web-based management group and the web-based management group were 71.6% and 82.6%, respectively. According to the aforementioned criteria, patients in the 2 groups were able to obtain effective anticoagulation overall. However, more patients in the web-based management group (n=210, 81.2%) received effective anticoagulation management than in the non–web-based management group (n=165, 63.5%). The Alfalfa app may improve the quality of anticoagulation through the following ways: (1) the app is equipped with patient education modules to improve patients' medical knowledge regarding anticoagulation; (2) the app is also equipped with useful medication and blood check reminder modules to prevent the occurrence of missed doses and prolong intervals between INR testing; and (3) the convenient model of anticoagulation management through the app is more acceptable to patients and can improve their medication compliance.

Of note, previous studies have reported a distribution roughly between 17.9% and 56.2% of TTRs under routine management [16] and that effective anticoagulant management is not available for 65% of patients [17]. The results of this study are not consistent with the aforementioned studies. This may be due to the relatively young sample composition of the non–web-based management group in this study, with an average age of 49 years, and their better compliance and medication-related knowledge. Additionally, China's health system construction in recent years has improved the availability of high-quality medical resources, which may also be the reason for this difference. Recently published studies have also reported that the TTR of patients in China under routine management mode exceeds 70% [18]. In terms of clinical events, more patients in the non–web-based management group experienced minor bleeding events during the 6-month follow-up period, but there was no statistically significant difference between the 2 groups for severe bleeding, thromboembolic events, and all-cause mortality. Web-based management can also increase the proportion of INR values in the therapeutic range and reduce the probability of INR values in the extreme subtherapeutic and extreme overtreatment ranges.

Chinese medical practitioners have extensively explored the remote management of warfarin. Numerous studies have demonstrated the benefits of remote management to varying degrees, although the specific approaches and conclusions of these studies are not exactly the same. Min Gu et al [19] conducted a randomized controlled study to evaluate the feasibility and clinical application effect of the “follow-up patient” mobile app in patients with atrial fibrillation taking warfarin, and the results showed that the anticoagulation management model guided by telemedicine technology could reduce the risk of minor bleeding and ischemic stroke and decrease the number of emergency department visits. Jinze Li et al [20] evaluated the security and efficacy of an “internet + smartphone app” in patients with mechanical heart valve replacement, and the TTR of the remote group was significantly higher than that of the non–web-based group, with no significant difference in the incidence of anticoagulation-related complications between the 2 groups during the 12-month follow-up period. Cao et al [3] conducted a retrospective, observational cohort study in 2018 comparing the efficacy and safety of anticoagulation management services provided by pharmacists through hospital anticoagulation clinics versus web-based anticoagulation clinics. In their study, patients in both groups had similar TTR and clinical event outcomes but a higher incidence of out-of-the-treatment range INR values in the web-based anticoagulation outpatient group.

Regarding the Alfalfa app used in this study, previous retrospective cohort studies have also shown that web-based administration improves TTR and reduces the incidence of minor bleeding events, major bleeding events, warfarin-related emergency department events, and warfarin-related hospitalizations [3]. In terms of the quality of anticoagulant management, the results of this study are consistent with the aforementioned study, in that remote administration can improve the quality of anticoagulation management in patients using warfarin. However, results in clinical events are not completely consistent and may be due to several factors. First, the study population came from different regions of China, and there were differences in demographic characteristics, dietary habits, adherence, and other factors, which in turn affected the clinical outcome of the anticoagulation therapy. Second, the existing studies had a follow-up period of 6 months to 1 year, and some studies lacked sufficient sample sizes (less than 100 people) to reflect the effect of remote anticoagulation management on clinical events with low incidences such as intracranial hemorrhage and ischemic cerebral infarction. It is expected that future large studies will demonstrate the long-term clinical outcomes of remote anticoagulation management.

Although new oral anticoagulants such as dabigatran and rivaroxaban have been marketed in China in recent years, they cannot completely replace warfarin clinically due to their specific indications and higher prices. Therefore, the warfarin management model still requires long-term exploration and optimization. As a new management model, web-based warfarin management has been positively evaluated by many medical practitioners. In addition to the clinical benefits, remote warfarin management is also beneficial to public health. First, using internet technology can reduce costs and increase patients' access to medical services [21]. Second, telemedicine can increase the efficacious use of medical and health resources and promote their redistribution in geographical distribution [22]. Moreover, the advantages of warfarin remote management are also evident in the transportation lockdown and social distancing measures taken to prevent the spread of COVID-19, in that patients no longer need to visit the hospital to obtain a dose adjustment regimen, reducing the risk of exposure to the novel coronavirus. Compared with urban areas in China, patients in rural areas face a long-term higher risk of intracranial hemorrhage and ischemic stroke and lack of effective anticoagulation management [23]. Benefiting from the efficient network infrastructure in China, the total number of internet users reached 1.011 billion by June 2021, of which the size of rural internet users reached 297 million [24]. These populations are likely to be beneficiaries of remote warfarin administration.

However, the use of internet technology has improved the quality of anticoagulation management while also raising the barriers to access to care, making the population of beneficiaries limited. Patients usually need to be trained in the use of the software before participating in warfarin telemanagement, which can be challenging for certain populations, such as older adults or those with low literacy levels [25]. For example, the usability evaluation findings of the Alfalfa app showed that some patients had difficulty using it, especially older adults [8]. There are 3 main reasons for this phenomenon. First, the user group was limited by age and literacy level, making it challenging for them to learn and use the telemedicine software. To solve this challenge, the users of the Alfalfa app are not limited to the patients themselves. Medical staff can encourage patients' family members to replace or assist this group with the operational steps of remote management. Second, the operation and functionality of the software are so complex that it takes a long time for patients to learn how to operate the software. Therefore, in the process of developing remote anticoagulation management software, medical staff need to fully communicate with software technicians to improve ease of use by simplifying the operation and adding suggestive symbols and text, thereby expanding the population that can benefit from remote anticoagulation management. Third, in addition to the basic operation of the app, patients need to acquire additional warfarin-related knowledge. This may not be directly related to the operation of the software, but it is important to ensure that patients are able to manage anticoagulation-related complications promptly and correctly. Striking a balance between the usability and security of telemedicine software is the focus of future research. Furthermore, data management and privacy protection are key issues in the implementation of telemedicine. Imprudent implementation of telemedicine technologies may lead to loss of patient privacy and autonomy, which telemedicine practitioners must carefully address.

### Study Limitations

This study has the following limitations. First, the inclusion process of patients was not randomized to groups and may have been subject to selection bias. Second, patients from western and northeastern China were not included in this study, and caution is needed to extrapolate the findings to the national level. Third, the 6-month follow-up period may make it difficult to capture the difference between web-based and non–web-based anticoagulation management in terms of low incidence of clinical events. Future studies with longer follow-ups will be needed to observe the long-term clinical outcomes of both management models.

### Conclusion

Compared with traditional non–web-based outpatient management, web-based management via the Alfalfa app may improve the quality of anticoagulation management of patients with respect to the percentage of patients meeting effective anticoagulation criteria. It can also reduce the incidence of minor bleeding events and the probability of INR values in the extreme subtherapeutic and extreme supratherapeutic ranges.

## Appendix

Multimedia Appendix 1 RECORD-PE (Reporting of Studies Conducted Using Observational Routinely Collected Health Data Statement for Pharmacoepidemiology) checklist.

Multimedia Appendix 2 Details of the Alfalfa app.

Multimedia Appendix 3 Occurrence of clinical events.

## Abbreviations

INR: international normalized ratio
ISTH: International Society on Thrombosis and Hemostasis
RECORD-PE: Reporting of Studies Conducted Using Observational Routinely Collected Health Data Statement for Pharmacoepidemiology
TTR: time in therapeutic range

## Data Availability

The data that support the findings of this study are available from the corresponding author upon reasonable request.

## References

[1] Barnes GD, Lucas E, Alexander GC, Goldberger ZD (2015). National trends in ambulatory oral anticoagulant use. Am J Med.

[2] Baumgartner H, Falk V, Bax JJ (2017). 2017 ESC/EACTS guidelines for the management of valvular heart disease. Eur Heart J.

[3] Cao H, Jiang S, Lv M, Wu T, Chen W, Zhang J (2021). Effectiveness of the Alfalfa app in warfarin therapy management for patients undergoing venous thrombosis prevention and treatment: cohort study. JMIR Mhealth Uhealth.

[4] Yao H, Zhan C, Sha X (2020). Current situation and distribution equality of public health resource in China. Arch Public Health.

[5] Yang X, Li Z, Zhao X, Wang C, Liu L, Wang C, Pan Y, Li H, Wang D, Hart RG, Wang Y, Wang Y (2016). Use of warfarin at discharge among acute ischemic stroke patients with nonvalvular atrial fibrillation in China. Stroke.

[6] Langan SM, Schmidt SA, Wing K, Ehrenstein V, Nicholls SG, Filion KB, Klungel O, Petersen I, Sorensen HT, Dixon WG, Guttmann A, Harron K, Hemkens LG, Moher D, Schneeweiss S, Smeeth L, Sturkenboom M, von Elm E, Wang SV, Benchimol EI (2018). The reporting of studies conducted using observational routinely collected health data statement for pharmacoepidemiology (RECORD-PE). BMJ.

[7] Xia X, Fu J, Wu T, Chen W, Jinhua Z (2019). Comparison of the outcomes of warfarin therapy and economics by online and offline anticoagulation management models: protocol for a randomised controlled trial. BMJ Open.

[8] Jiang S, Lv M, Wu T, Chen W, Zhang J (2022). A smartphone application for remote adjustment of warfarin dose: Development and usability study. Appl Nurs Res.

[9] McDowell Tzu-Yun, Lawrence John, Florian Jeffry, Southworth Mary Ross, Grant Stephen, Stockbridge Norman (2018). Relationship between International Normalized Ratio and Outcomes in Modern Trials with Warfarin Controls. Pharmacotherapy.

[10] Bowman T S, Gaziano J M, Kase C S, Sesso H D, Kurth T (2006). Blood pressure measures and risk of total, ischemic, and hemorrhagic stroke in men. Neurology.

[11] Hilkens Nina A, Greving Jacoba P, Algra Ale, Klijn Catharina J M (2017). Blood pressure levels and the risk of intracerebral hemorrhage after ischemic stroke. Neurology.

[12] Schlunk Frieder, Chang Yuchiao, Ayres Alison, Battey Thomas, Vashkevich Anastasia, Raffeld Miriam, Rost Natalia, Viswanathan Anand, Gurol M Edip, Schwab Kristin, Greenberg Steven M, Rosand Jonathan, Goldstein Joshua N (2016). Blood pressure burden and outcome in warfarin-related intracerebral hemorrhage. Int J Stroke.

[13] Schulman S, Kearon C, Subcommittee on Control of Anticoagulation of the ScientificStandardization Committee of the International Society on ThrombosisHaemostasis (2005). Definition of major bleeding in clinical investigations of antihemostatic medicinal products in non-surgical patients. J Thromb Haemost.

[14] Zhang J, Liu M, Chen Q, Wu J, Cao H (2017). Outcomes of an online pharmacist-managed anticoagulation clinic for individuals on warfarin therapy living in rural communities. Thromb Res.

[15] Hart RG, Pearce LA, Aguilar MI (2007). Meta-analysis: antithrombotic therapy to prevent stroke in patients who have nonvalvular atrial fibrillation. Ann Intern Med.

[16] Feng X, Huan Y, Lv Y (2015). Letter by Feng et al regarding article, “Ischemic stroke and intracranial hemorrhage with aspirin, dabigatran, and warfarin: impact of quality of anticoagulation control”. Stroke.

[17] Li X, Pathadka S, Man KKC, Ng VWS, Siu CW, Wong ICK, Chan EW, Lau WCY (2020). Comparative outcomes between direct oral anticoagulants, warfarin, and antiplatelet monotherapy among Chinese patients with atrial fibrillation: a population-based cohort study. Drug Saf.

[18] Jiang S, He Q, Yan J, Zhao L, Zheng Y, Chen P, Chen X (2021). Evaluation of a pharmacist-led remote warfarin management model using a smartphone application (Yixing) in improving patients' knowledge and outcomes of anticoagulation therapy. Front Pharmacol.

[19] Gu M (2019). Study of telemedicine application for warfarin anticoagulation management in atrial fibrillation. Thesis. Yangzhou University.

[20] Li JZ, Dong Y, Qian Y (2019). Remote management of anticoagulation after cardiac mechanical valve replacement:a prospective cohort studyJ. Chinese Journal of Clinical Thoracic and Cardiovascular Surgery.

[21] Baksheev A, Turchina Z, Sharova O (2020). Innovations in medicine: features of regulation and prospects for the development of telemedicine. Revista Inclusiones.

[22] Cui F, Ma Q, He X, Zhai Y, Zhao J, Chen B, Sun D, Shi J, Cao M, Wang Z (2020). Implementation and application of telemedicine in China: cross-sectional study. JMIR Mhealth Uhealth.

[23] Wang W, Jiang B, Sun H, Ru X, Sun D, Wang L, Wang L, Jiang Y, Li Y, Wang Y, Chen Z, Wu S, Zhang Y, Wang D, Wang Y, Feigin VL (2017). Prevalence, incidence, and mortality of stroke in China. Circulation.

[24] (2021). The 48th statistical report on the development of the Internet in China. China Internet Information Center.

[25] Correa T, Pavez I (2016). Digital Inclusion in rural areas: a qualitative exploration of challenges faced by people from isolated communities. J Comput-Mediat Comm.

