# Supplementary material for: Web-Based Warfarin Management (Alfalfa App) Versus Traditional Warfarin Management: Multicenter Prospective Cohort Study
Source: J Med Internet Res. 2024 Jul 29;26:e46319. doi: 10.2196/46319 (PMC11319884; doi:10.2196/46319)
Supplement: Multimedia Appendix 2 [file jmir_v26i1e46319_app2.docx]

**Introduction of remote management tools (Alfalfa App)**

The Alfalfa App consists of a backend management system and an anticoagulation management system based on the WeChat public platform. The latter can be divided into Alfalfa health management on the patient side and Alfalfa anticoagulation guidance on the medical side. The primary function of the Alfalfa App is to enable the interaction of information between patients and medical personnel, enabling patients to inform their doctors or pharmacists of changes in coagulation results and health status via the Internet. The patient can then take the medication according to the dosage adjustment protocol and the relevant medical advice from the doctor or pharmacist and take the next INR test on time. During the design process of Alfalfa App, clinical pharmacists specializing in anticoagulation, doctors from relevant departments and software engineers repeatedly discussed and determined the target population, design principles and main functions of the software. The Alfalfa App has been evaluated for ease of use ^[8]^, and the results show that the ease of use of the medical side of the Alfalfa App is good, but the ease of use of the patient side needs to be improved.

**1.1 Patient-side functions**

The functions of the patient side of Alfalfa App include four modules: remote medication consultation, medication and blood test time reminder, warfarin knowledge popularization, and anticoagulation community. The remote medication consultation module uploads INR results, warfarin dose and information related to diet and physical condition, and receives medical advice from medical staff in reply to enable remote management of warfarin. In the medication and blood test time reminder module, patients can set the time and frequency of medication reminder according to their own medication habits. In addition, the module is able to remind patients to review their INR values two days before the date of the next coagulation test, as replied by the medical staff. The warfarin knowledge science module publishes content on warfarin dosing precautions and cardiovascular disease-related knowledge, including the significance of INR, factors influencing warfarin efficacy, and disease characteristics of heart valve disease, atrial fibrillation, and stroke. The Anticoagulation Community module serves as a platform for direct patient-patient and patient-medical staff dialogue and communication. Patients can share their experience of taking warfarin in the anticoagulation community, and they can also ask various questions, such as how to use the Alfalfa App and the principles of handling adverse reactions. The doctor reply screen and the home page of the patient side can be seen in Figure 1.


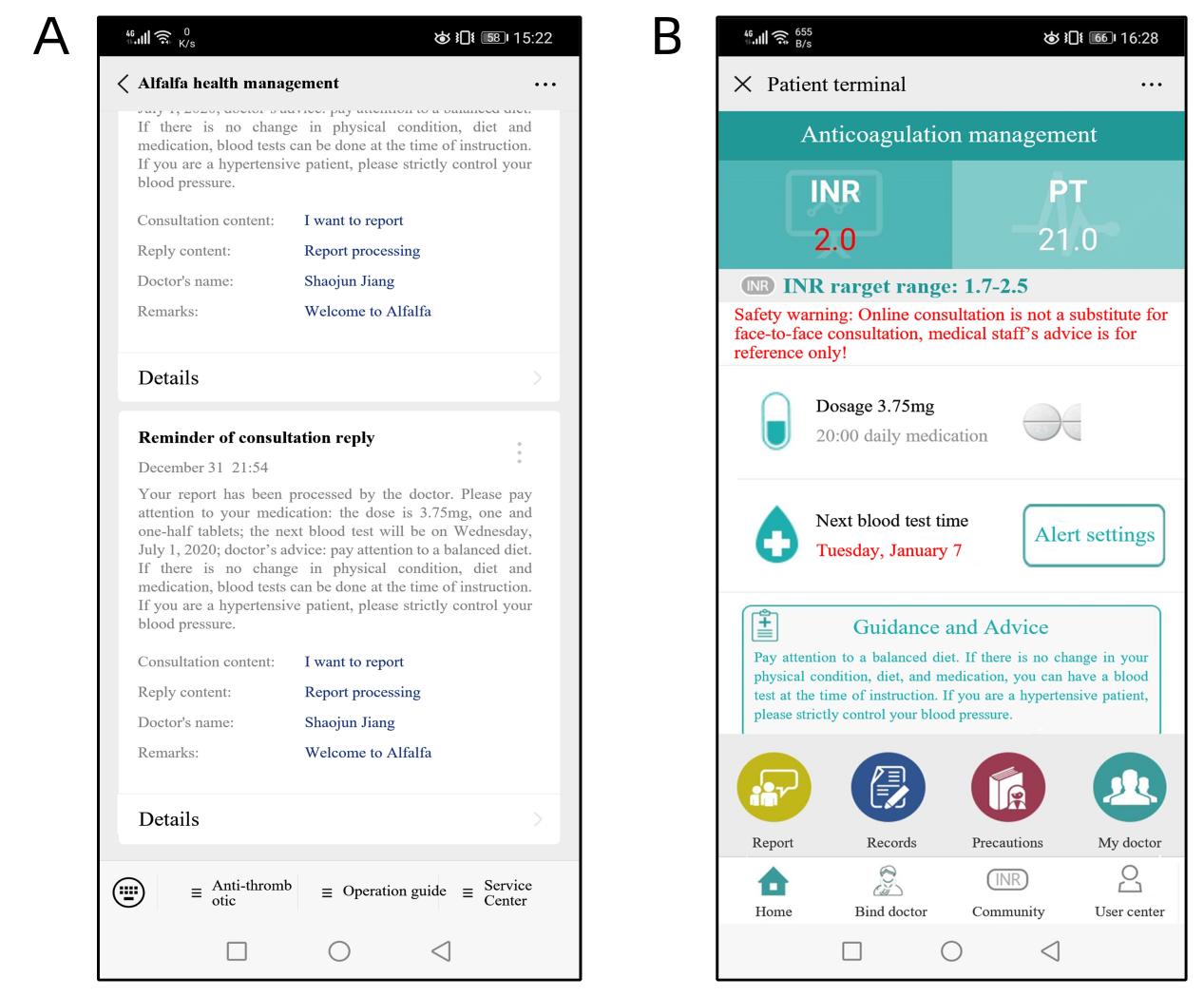


Figure 1. Doctor’s Reply (A) and Home Page of Patient Terminal (B)

**1.2 Medical side functions**

Remote dose adjustment is the core function of the medical side of Alfalfa, ensuring that the doctor or pharmacist can make dose adjustment plans based on patient reports and, if necessary, check the patient's details and previous reporting history. In addition, the Alfalfa App is equipped with an algorithm that analyzes how well the patient's target INR range matches the current reported results and automatically develops an initial dose adjustment and timing plan for the next blood test. Medical staff can make changes to the protocol based on their own clinical experience or the actual situation of the patient. Smart revision of medical advice means that the Alfalfa App can automatically revise medical advice based on patient information or the dose adjustment plan developed. For example, the Alfalfa App will automatically remind patients to strictly monitor and control their blood pressure when developing dose adjustment protocols for patients with comorbid hypertension. When the dose set by the doctor is 0 mg, the doctor's order will be automatically adjusted to "stop taking warfarin tonight for 2 days, check blood on day 3 and report". Figure 2 shows the home page of the medical side of the Alfalfa App and the interface for processing reported information.


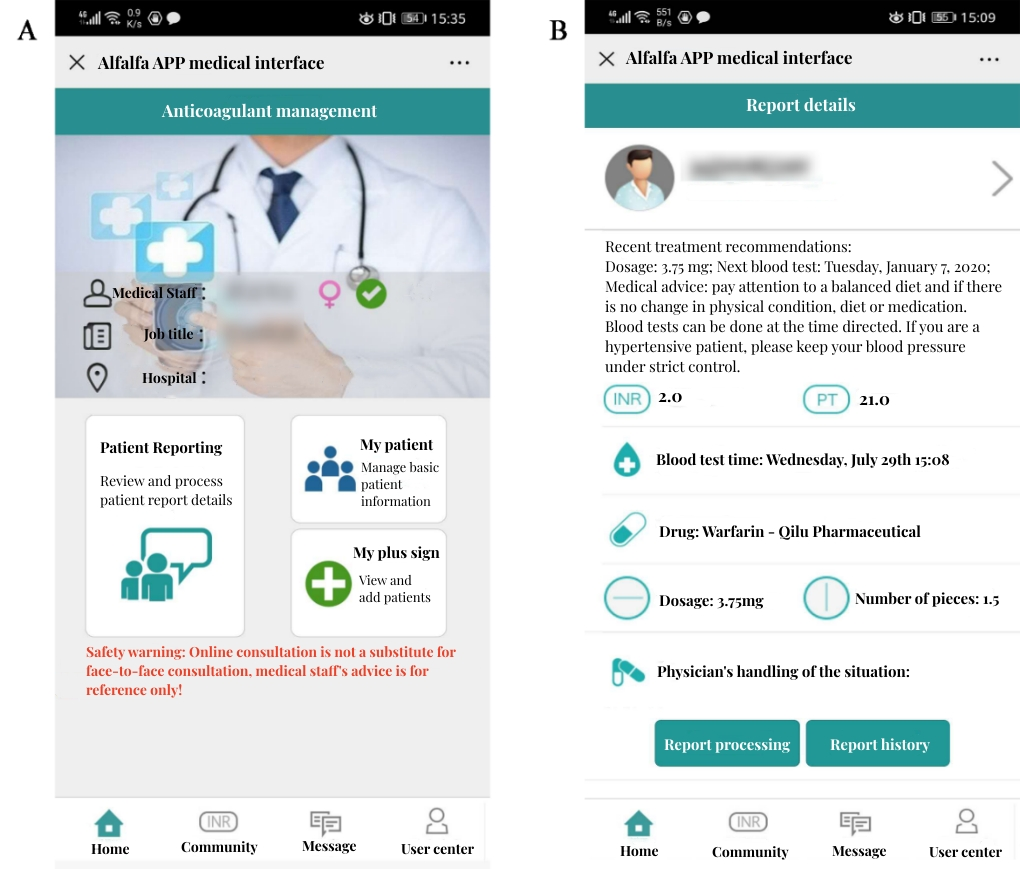


Figure 2. Home Pageof Doctor Terminal (A) and Processing the Reported Record (B)

**1.3 Other auxiliary functions**

In order to reduce the incidence of anticoagulation-related clinical events, the following ancillary features have been added to the Alfalfa App. INR extreme value warning and blood pressure management. As far as the efficacy aspect of warfarin is concerned, transient INR values below the target range do not increase the risk of thromboembolism.^[9]^ In contrast, INR >4 significantly increases the incidence of cerebral hemorrhage.^[10]^ Furthermore, the percentage of time with an INR >4.5 was positively associated with the risk of major bleeding.^[11]^ Therefore, prompt intervention is needed for patients presenting with very high INR values, while intervention when INR is below the therapeutic range does not appear to be essential. The INR extremes warning feature in the Alfalfa App will automatically pop up on the screen when a patient reports an INR value above 4.5 with the warning "Your INR value is too high, please go to the hospital immediately".

Intracranial hemorrhage is one of the serious adverse effects of warfarin, and numerous studies have confirmed that hypertension is closely associated with the incidence of intracranial hemorrhage ^[11,12]^. For patients taking warfarin, blood pressure management is even more important. Therefore, hypertensive patients are asked to report their current blood pressure while uploading INR results so that physicians can guide patients to control their blood pressure and reduce the occurrence of adverse reactions.
