# Supplementary figures and images for: Web-Based Warfarin Management (Alfalfa App) Versus Traditional Warfarin Management: Multicenter Prospective Cohort Study
Source: J Med Internet Res. 2024 Jul 29;26:e46319. doi: 10.2196/46319 (PMC11319884; doi:10.2196/46319)

**
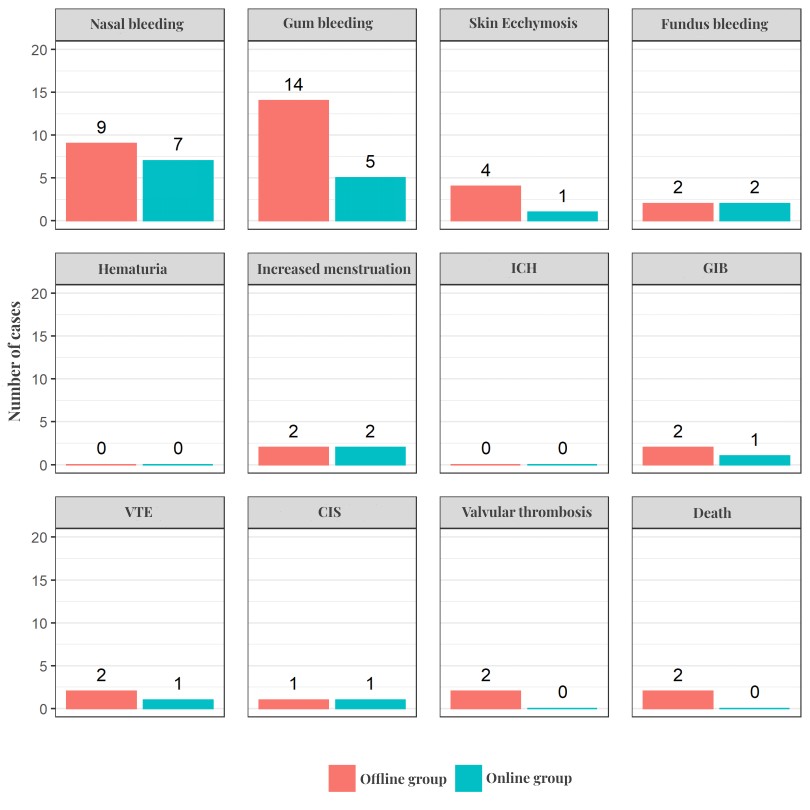
**

**Figure 3. Composition of clinical events**

Supplement: Multimedia Appendix 3 [file jmir_v26i1e46319_app3.docx]
